# Reconstruction of the distal radioulnar joint with rib perichondrium – midterm follow-up

**DOI:** 10.1186/s12891-022-05335-4

**Published:** 2022-04-26

**Authors:** Daniel Muder, Torbjörn Vedung

**Affiliations:** 1 Department of Surgical Sciences/Orthopedics & Hand Surgery, Uppsala University, Entrance 70, 1st floor, 751 85 Uppsala, Sweden; 2 Department of Orthopedics, Falu Lasarett, Lasarettsvägen 10, 791 82 Falun, Sweden; 3 Elisabeth Hospital, Aleris Healthcare AB, Geijersgatan 20, 752 26 Uppsala, Sweden

**Keywords:** Perichondrium, Distal radioulnar joint, Reconstruction, Arthritis, Transplantation, Cartilage

## Abstract

**Background:**

Reconstruction of an osteoarthritic distal radioulnar joint (DRUJ) in patients with high physical demands and a long lifetime expectancy is challenging. A variety of methods like implant surgery and salvage procedures as partial or total ulnar head resection and the Sauve-Kapandji procedure are reasonable options in the elderly patient but not in young individuals since it often compromises manual power and stability and may cause impingement problems. Reconstruction of the DRUJ with rib perichondrium is a new treatment option with promising short-term outcome. The aim the present study was to investigate if the outcome is consistent over time.

**Methods:**

Four female patients with a mean age of 40.5 years suffered severe unilateral osteoarthritis in the DRUJ. They underwent reconstruction of the joint with rib perichondrium transplants. Preoperatively, mean pain under manual load was 8.5 (range 7–10) and 4.2 (range 2–5) at rest, using the visual analogue scale (VAS). Range of motion (ROM) in forearm rotation was on average 118° and grip strength was 86% in comparison to the contralateral hand. The outcome was assessed at a clinical follow-up in 2016, measuring ROM, grip-strength, pain at rest and under manual load and DASH-score. Radiological examination was performed. An additional follow-up by letter was performed in 2021 using a patient-reported-outcome survey (PROS). The patients were asked to grade the ROM and grip-strength as changed or unchanged in comparison to the clinical follow-up in 2016.

**Results:**

At clinical follow-up at a mean of 3.1 years (range 1–5) after surgery, pain level had decreased to VAS 1.5 (0–5) under load and all patients were pain free at rest. Forearm rotation was on average 156° (range 100–180) and grip strength was 97% of the unoperated hand. The mean DASH-score was 14.4 (0–45). An additional follow-up by letter was conducted at a mean of 7.5 years (5.5–9.5) after surgery. ROM and grip strength were reported as unchanged by all patients in relation to the previous clinical follow-up. No additional surgery or complications were reported.

**Conclusion:**

Reconstruction of the osteoarthritic DRU-joint with rib perichondrium transplantation can provide good clinical outcome with perseverance over time.

**Level of evidence:**

IV.

## Background

Surgical options regarding treatment of osteoarthritis in the distal radioulnar joint (DRUJ) are limited and remain a challenging task, especially in young non-rheumatoid patients with high physical demands and with an expected remaining lifetime of 30–50 years or longer. Most of the established surgical methods cause significant changes of the bony anatomy, e.g. total (Darrach) or partial (Bowers) resection of the ulnar head, and the Sauve-Kapandji procedure [1]. The resulting condition after these traditional procedures may cause painful impingement between the distal ulnar stump and the radius during manual load [2] and are commonly used in older individuals with rheumatoid changes [3]. In the Bowers procedure, sufficient soft tissue surrounding the joint is mandatory in order to avoid stylocarpal impingement, which is a potential complication [3]. In the literature the complication rate has been reported to vary considerably, between 14–44% [3, 4]. High complication rates have been described for both the Darrach (30%) and the Sauve-Kapandji (50%) procedure [2, 5]. The results after implant arthroplasty have improved but there are still problems with restricted range-of-motion, persistent pain, implant loosening and instability. This may result in impaired grip strength of 73% of the contralateral side [6] and a high reoperation rate of 29% [7]. One of the most common implants is the ulnar head replacement prothesis [8]. The midterm survival rate has been reported as high as 90–100% [9, 10], but outcomes scores in the same studies indicated substantial residual disability. Revision surgery secondary to any of the methods described above are challenging and the outcome unclear. The significant physical impairment after a total DRUJ fusion is not acceptable for most patients, and therefore rarely used.

In this context reconstruction of the DRUJ with autologous tissue in combination with efforts to preserve most of the bony anatomy of the joint is a reasonable approach which has been performed previously [11]. Perichondrium from the rib has shown chondrogenic potential in animal experiments [12–16] and has been used to resurface finger joints since the 1970’s [17–19]. In a recent study, perichondrial grafts from the rib to the metacarpophalangeal (MCP) and the proximal interphalangeal (PIP) joints showed encouraging long-time survival in comparison to modern surface replacement implants [20]. In 2014, the perichondrium transplantation technique was adapted to the DRUJ by the senior author of this paper [11]. The short-term follow up results were promising regarding pain, grip-strength and ROM. The positive results encouraged us to proceed with two additional patients with similar problems. The purpose of the present study was to investigate the mid-term outcome after perichondrium transplantation to the DRUJ. Are the previously reported short-term results consistent over time?

## Methods

We identified all patients that underwent surface reconstruction to both sides of the DRUJ with rib perichondrium transplants at Uppsala University hospital between 2011–2016. The patients agreed to participate in the study by written informed consent.

### Characteristics of the study population (see Table 1for details)

**Table 1:** Pre- and postoperative data

Case	Year of surgery	Motion/Strength/ Pain	Pre- operative	Follow up 1 2012^b^	Follow up 2 2016 (clinical, mean 3.1y)	Follow up 3 2021 (by letter, mean 7.5y)
Case 1	2011	Supination	70	90	90	Unchanged^a^
Case 1		Pronation	50	75	90	Unchanged^a^
Case 1		Flexion	70	75	75	Unchanged^a^
Case 1		Extension	75	75	75	Unchanged^a^
Case 1		JAMAR (KG)	36	38	33	Unchanged^a^
Case 1		Pain (VAS)	9	0	0	1
Case 1		DASH	77	4	0	6.7
Case 2	2013	Supination	70	40	85	Unchanged^a^
Case 2		Pronation	70	80	80	Unchanged^a^
Case 2		Flexion	75	70	75	Unchanged^a^
Case 2		Extension	60	75	75	Unchanged^a^
Case 2		JAMAR (KG)	26	27	28	Unchanged^a^
Case 2		Pain (VAS)	9	3	1	1
Case 2		DASH	77	27	5	0
Case 3	2014	Supination	30	––	50	Unchanged^a^
Case 3		Pronation	25	––	50	Unchanged^a^
Case 3		Flexion	60	––	60	Unchanged^a^
Case 3		Extension	65	––	65	Unchanged^a^
Case 3		JAMAR (KG)	13	––	21	Unchanged^a^
Case 3		Pain (VAS)	7	––	5	5
Case 3		DASH	––	––	45	23.3
Case 4	2016	Supination	80	––	90	Unchanged^a^
Case 4		Pronation	80	––	90	Unchanged^a^
Case 4		Flexion	75	––	75	Unchanged^a^
Case 4		Extension	75	––	75	Unchanged^a^
Case 4		JAMAR (KG)	24	––	22	Unchanged^a^
Case 4		Pain (VAS)	9	––	0	3
Case 4		DASH	––	––	7.5	15
Mean of all Cases		Supination	62.5	––	79	Unchanged^a^
		Pronation	56	––	77.5	Unchanged^a^
		JAMAR (KG)	25	––	26	Unchanged^a^
		Pain (VAS)	8.5	––	1.5	2
		DASH	––	––	14.4	11.3

^a^Follow-up 2021 by letter, Clinical values reported by the patients as changed/unchanged compared to the latest follow-up in 2016

^b^The figures in column 5 are previously published in reference [11]. Reprinted with permission

The study cohort consists of four female patients with a mean age of 40,5 years (range 37–47) at the time of surgery. All four had significant unilateral osteoarthritis in the DRUJ. Patient 1–3 had no history of any previous trauma. Patient 1–2 had an ulna minus, while patient 3 had an ulna plus, which previously had been leveled with an ulna shortening osteotomy. Patient 4 suffered a distal radius fracture as a child and went through an ulna shortening osteotomy as an adult. The DRUJ became unstable, the TFCC was reinserted to the fovea using open transosseous suture technique. The DRUJ regained stability but subsequently developed osteoarthritis. In the study cohort, pain under manual load was on average graded to 8,5 (range 7–9), using the visual analogue scale (VAS). Pain at rest was on average graded to VAS 4,25 (range 2–5). The preoperative ROM in the DRUJ was severely impaired in case 3 with supination/pronation figures of only 30/25 degrees, while the same figures in the other three patients was only mildly decreased (mean 73/67 degrees).

### Clinical examination

**Fig. 1:**
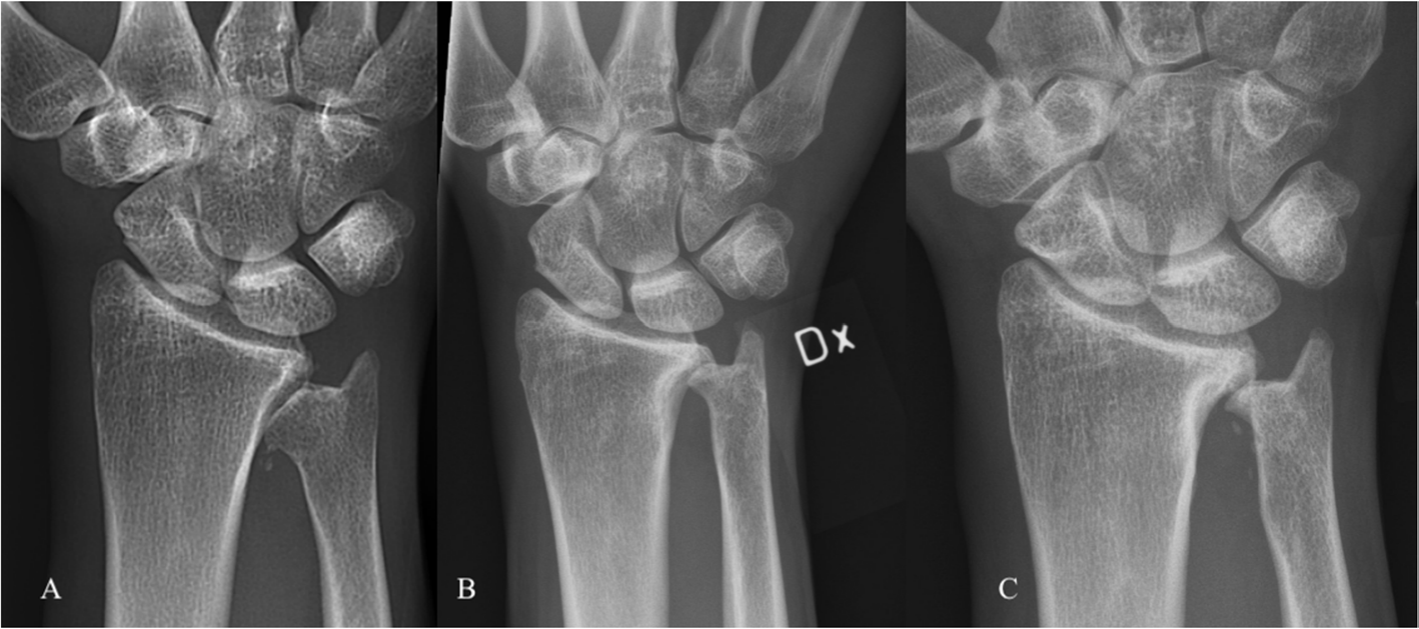
Posterior-anterior (PA) view with conventional radiography (CR) in case 1 with preoperative signs of osteoarthritis in the DRUJ (**A**). An increased distance in the DRUJ at 2.5 years follow-up (**B**), and similar findings 5 years postoperatively (**C**)

**Fig. 2:**
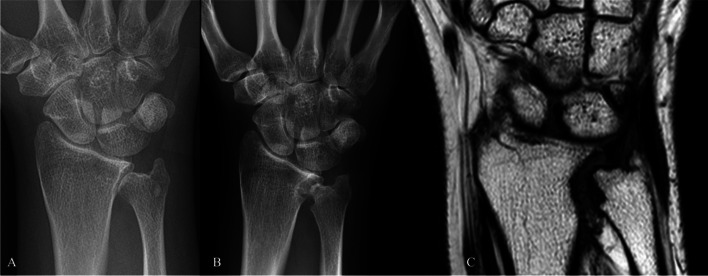
PA radiographs in case 2 showing a reduced joint space in the DRUJ preoperatively (**A**). At 1 year follow-up, some of the subchondral bone appears uneven but the distance between radius and ulna is wide (**B**). MRI 2 years postoperatively revealed similar findings (**C**)

**Fig. 3:**
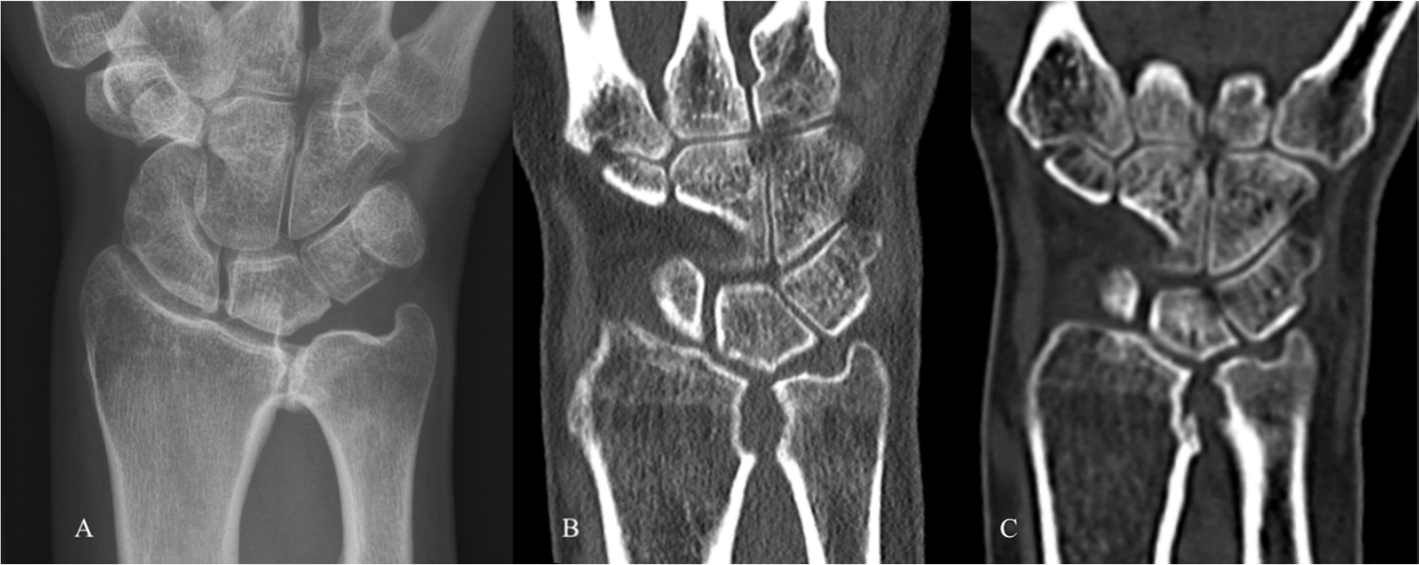
PA radiographs in case 3 with osteoarthritis in the DRUJ (**A**). Computer tomography (CT) at 6 months follow-up showing a wide distance in the joint (**B**), which is unchanged 5 years postoperatively (**C**)

**Fig. 4:**
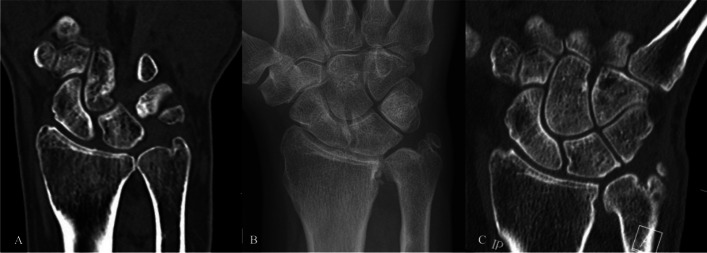
Preoperative CT in case 4 with an obliterated joint space in the DRUJ (**A**). Postoperative CR with an increased distance in the joint (**B**), which is confirmed with CT 2.5 years postoperatively (**C**)

The outcome was assessed at a clinical follow-up in 2016, measuring the wrist and forearm range-of-motion (ROM), grip-strength (JAMAR, Sammons Preston Inc., Bolingbrook, IL, USA, average of three consecutive measurements), pain at rest and under manual load (VAS) and DASH-score (Disabilities of the Arm, Shoulder, and Hand). Radiological examination was performed at various postoperative occasions (Fig. 1, 2, 3 and 4). An additional follow-up by letter was performed in 2021 using a patient-reported-outcome survey (PROS). The patients were asked to grade the present ROM and grip-strength as *changed* or *unchanged* in comparison to the clinical follow-up in 2016. In addition, DASH-score and pain at rest and under manual load (VAS) was included in the survey. The short-term results in the two initial cases were reported in 2014 [11], and these figures are included in Table 1 in order to facilitate comparisons over time.

### Surgical procedure

The technique for harvesting rib perichondrium has previously been described in detail [11, 17, 20]. The harvest is made through an incision in the sub-mammary crease and the perichondrium from the 6^th^ or 7^th^ rib is peeled off the cartilage from the bone-cartilage junction to the sternum. The skin incision must stop at the medial margin of the sub-mammary crease to avoid unsightly scarring. The harvest medial to this point is made in a subcutaneous fashion. In order to gain access to the sigmoid notch, the ulnar head and to facilitate proper attachment of the grafts, a dorsal and a volar approach is needed. The dorsal approach is made through a curved or zigzag incision, the fifth extensor compartment is incised longitudinally and the extensor digiti minimi tendon is retracted radially. The floor of the tendon sheet is incised longitudinally and the sixth extensor compartment is retracted in ulnar direction in a subperiosteal fashion to expose the DRUJ. Great care is taken to avoid injury to the triangular fibrocartilage complex (TFCC) and to leave the sixth extensor compartment intact. The volar approach is made by an incision just radial to, and in parallel with, the flexor carpi ulnaris (FCU) tendon. The ulnar nerve and artery are identified and carefully retracted ulnarly, while the finger flexor tendons are retracted radially. The DRUJ capsule is incised longitudinally to expose the joint, while most of the pronator quadratus muscle is left intact. The TFCC is identified and carefully protected from the volar access as well. The eroded joint surfaces are removed down to bleeding subchondral cortex. The anatomical shape and curvature of the sigmoid notch and the ulnar head must be preserved. The resection can be made by an air-driven oscillating chisel or by hand with a curved chisel. A thin layer of subchondral cortex should be left intact to provide a solid and stable recipient site. The resulting secondary defect after preparation of the recipient site is about 2 mm in depth, on each side of the joint.

Perichondrium graft harvested from one rib is usually long enough, about 5 cm, to cover both joint surfaces. About one third of the graft is often sufficient to cover the sigmoid notch, and two thirds of the graft is long enough to cover the ulnar head. If more graft is needed, another transplant can be harvested from a neighboring rib. The thickness of the perichondrium graft is about 1 mm. The inner layer of the perichondrium (the cambium layer) that has been in contact with the rib cartilage should be placed facing the joint space, while the fibrous outer layer of the perichondrium is placed towards the recipient site. The grafts are secured by osteosutures both volarly and dorsally using drill holes and resorbable 3.0 sutures. The attachment is reinforced with a layer of fibrin glue (TISSEEL, Baxter Healthcare Corporation, Westlake, CA, USA) placed underneath the graft before tying the last osteosutures. Gentle pressure is applied over the joint for a few minutes, and eventual excess of glue is removed. A thin Silicone sheet (0.5 mm, Atos Medical AB, Hörby, Sweden) is positioned in between the grafts to avoid adhesions (chondrodesis)**.** The sheet should be secured with sutures to either the dorsal or the volar capsule in order to stay in place and facilitate later removal. On the dorsal side, the connection between the sixth extensor compartment and the TFCC is reconstructed with absorbable sutures, the fifth extensor compartment is restored with the extensor digiti minimi tendon in anatomical position. Volarly, the capsule incision is closed with absorbable sutures. No shortening or extra tension in the closing of the capsule is normally needed. A long cast over the forearm and elbow is applied to avoid forearm rotation during the first 4 postoperative weeks. The silicone sheet is removed after about 2 months through a small volar or dorsal incision under local anesthesia. The sheet is grabbed with a forceps and pulled out.

## Results

The mean follow-up time for the clinical assessment in 2016 was 3.1 years (1-5y). The pain level had decreased to VAS 1.5 (0–5) under load and all patients were pain free at rest. Total ROM in forearm rotation had increased and was nearly normal (mean 156 degrees, range 100–180) and the grip-strength was 97% in comparison with the non-operated side (range 76–122%). None of the patients had any postoperative instability in the DRUJ. The mean follow-up time for the additional PROS in 2021 was 7.5 years (5.5–9.5y). The ROM and grip strength was reported as unchanged by all patients. Two of the patients reported a slight elevation of pain-level in comparison to the clinical assessment in 2016. The average pain level had increased to VAS 2.5 (1–5). The mean DASH-score was 14.4 (0–45) at the 3.1-year follow-up, and 11.3 (0–23.3) at the 7.5-year follow-up. No additional surgery or complications were recorded or reported. None of the patients experienced any donor site morbidity (see Table 1 for details).

## Discussion

The mid-term outcome after reconstruction of the DRUJ with rib perichondrium is promising and the results seem to be consistent over time.

It is hard to find reliable surgical solutions to reconstruct painful osteoarthritic DRUJs in young non-rheumatoid patients. In the long-term, complications tend to occur which may cause hesitation to intervene surgically in these patients. Partial or total resection of the ulnar head are reasonable treatment options in the elderly patients with rheumatoid changes, but hardly for young non-rheumatoid patients. The Darrach procedure is hampered with problems as instability and painful impingement of the remaining ulnar stump towards the radius [1]. In a recent patient-reported-outcome study by Eberlin, complication and reoperation rates after the Darrach procedure (*n* = 57) and the Sauve Kapandji procedure (*n* = 28) were studied. The complication rate was reported to be 30% and 50% respectively, while the reoperation rate was 18% and 36% respectively. Overall, 52 patients (61%) in this study completed a PROS, and the authors did not find any significant difference in pain and satisfaction rate in between the two groups [2]. In a recent long-term follow-up study of the Sauve-Kapandji procedure, Nagy et al. found a high incidence of revision surgery due to instability to the proximal ulnar stump and recommended a restriction of this method to only very selected cases [5]. If the surrounding soft tissue is sufficient, the clinical outcome has been reported to be reasonably good after the Bowers procedure in both rheumatoid and none-rheumatoid patients by several authors [3, 21–23]. The method is considered as a salvage procedure [21, 24] and the main ambition with this technique, as described by Bowers in 1985, is to reduce pain and to improve the ROM in the DRUJ, not to stabilize the joint [25]. In this context the method is suitable for the rheumatoid patients, as these patients usually have lower physical demands in comparison to the non-rheumatoid patients. Several modifications of the procedure has been reported, as interposing a tendon or a flap of the extensor retinaculum into the joint, or dorsalisation of the extensor carpi ulnaris, in efforts to increase stability to the ulnar stump and achieve better results also in the non-rheumatoid patient[22, 26–28]. A relatively high DASH score (31 and 35 respectively) has been reported in combination with a good patient reported outcome measure (PROM) after the Bowers procedure [3, 21]. In a recent report by Nawijn, the relatively high DASH score in relation to low pain and high satisfaction rate might be attributed to the fact that the DASH score reflects not only the DRUJ problem but also general problems in the wrist caused by inflammatory arthritis or posttraumatic sequelae [3].

The salvage options after a failure, following a Darrach or Sauve Kapandji procedure, is mainly limited to implant surgery of some kind. In general, the results after implant surgery to the DRUJ has improved during the last decade. However, implants often fail to achieve function suitable for heavy load in the long term. Restricted range-of-motion, persistent pain and implant loosening are common problems[6, 7, 10].

The surgical method to resurface the DRUJ with rib perichondrium was reported in 2014 along with the short-term results of the first two patients in the present cohort [11]. The gratifying outcome in these patients have persisted over time, and actually improved in case 2, resulting in an almost normal function in the reconstructed joints. The follow-up time for the two additional patients is shorter but the results are similar, especially in the fourth case. The third case still has problems with pain and restricted ROM but the results have clearly improved in comparison to the preoperative findings. The preoperative problems in the third case differed somewhat in comparison to the others as the main problem was an impaired ROM. The results in the additional PROS (by letter) showed persistence in the outcome with an unchanged ROM and grip-strength in all cases, and only a slight increase in pain (VAS) in two cases (from 0 to 1 in the 1^st^ case, and from 0 to 3 in the 4^th^ case). The changes in DASH-score, with a decrease in half and an increase in the other half of the study group, is difficult to interpret as the DASH-score might be influenced by many things (e.g. other problems with the arm/hand). In a recent paper, the long-term outcome (mean 37 years) after perichondrium transplantation to the metacarpophalangeal (MCP) joint and the proximal interphalangeal (PIP) joint was presented [17]. Three early failures were reported, while the remaining eleven patients in the study-cohort had no additional surgery after the joint reconstruction almost four decades earlier. The authors suggested that function of the resurfaced joints will remain favorable in the long-term in most patients with favorable short-term outcome.

There is often a contrast in between the clinical outcome and the radiological appearance after a perichondrium transplantation [11, 17, 29]. In analogy with previous reports, we found radiological signs of bone resorption and remodeling over time in all four cases (Figs. 1, 2, 3 and 4). The reconstructed joint will not look normal on radiographs. The gap between the sigmoid notch and the ulnar head will be wider. This may be explained by hypertrophy and thickening of the grafts, filling the gap in the joint. In a recent rat study, rib perichondrium was transplanted to cover a localized full-thickness articular cartilage defect created in the rat knee. A relatively high proliferation rate was found early after the transplantation followed by a later increase in cell size [12]. The grafts produced hyaline cartilage that filled out the defects and subsequently differentiated to achieve a chondrocyte marker expression pattern and structure similar to the surrounding articular cartilage [12].

### Limitations and strength

It is a clear limitation that the study group only consisted of four patients. The retrospective study design, and the lack of a comparative study group representing another surgical method (e.g. implant surgery or the Bowers hemi-resection procedure), makes it hard to draw any definite conclusions. The mean age in the present study is relatively young (40.5 years) in comparison to most studies about surgery towards osteoarthritis in the DRUJ. Moreover, all four were non-rheumatoid patients. A longer follow-up time for the clinical assessment (mean 3.1 years) would have strengthen the study. The additional survey sent by letter was an effort to overcome this drawback during the ongoing COVID-19 pandemic. In addition, if the pattern of the findings in the recent long-term follow up after perichondrium transplantation to the MCP and the PIP joints [17] is a consistent feature, most failures probably appear at an early postoperative stage. A larger study-cohort and longer follow-up time is needed to conclude if this surgical technique is superior to the more traditional methods or not. A prospective randomized study comparing perichondrium transplantation with implant surgery, or the Bowers procedure would have been helpful but is most likely not feasible due to ethical matters, related to the second surgical site at the ribcage.

## Conclusion

Reconstruction of osteoarthritic DRUJ surfaces with rib perichondrium is a novel technique providing good clinical results at mid-term follow-up. The method is worth consideration, especially in young non-rheumatoid patients with high physical demands and a long remaining life expectancy. It is important that the soft tissue surrounding the joint is relatively intact. Preoperative stability in the DRUJ is mandatory and the range of motion should, in an ideal situation, be almost normal. Cases with severe scarring should be avoided as the technique requires surgical access both volarly and dorsally. It preserves the soft tissue and most of the bone stock, enabling eventual revision surgery or implant surgery later in life.

## Data Availability

The data set supporting the conclusion of this article is available on request to the corresponding author.

## Abbreviations

DRUJ: Distal radioulnar joint
VAS: Visual analogue scale
ROM: Range of motion
DASH: Disabilities of the arm, shoulder and hand
MCP: Metacarpophalangeal
PIP: Proximal interphalangeal
PROS: Patient reported outcome survey
TFCC: Triangular fibrocartilage complex
PROM: Patient rated outcome measure
CR: Conventional radiography
CT: Computer tomography
MRI: Magnet resonance imaging
PA: Posterior-anterior
